# *IL-6*和*IL-1β*单核苷酸多态性与非吸烟女性肺癌易感性的关系

**DOI:** 10.3779/j.issn.1009-3419.2014.08.06

**Published:** 2014-08-20

**Authors:** 濛 宿, 宝森 周

**Affiliations:** 1 110042 沈阳，辽宁省肿瘤医院 Liaoning Cancer Hospital & Institute, Shenyang 110042, China; 2 110001 沈阳，中国医科大学流行病学教研室 Department of Epidemiology, China Medical University, Shenyang 110001, China

**Keywords:** 女性肺癌, *IL-6*基因, *IL-1β*基因, 基因多态性, 易感性, Female lung cancer, *IL-6* gene, *IL-1β* gene, Gene polymorphism, Susceptibility

## Abstract

**背景与目的:**

白介素-6(interleukin-6, IL-6)和白介素-1β(IL-1β)是白介素家族中具有多功能生物活性的细胞因子。IL-6-634(rs1800796)和IL-1β-31(rs1143627)分别是*IL-6*基因和*IL-1β*基因上的两个常见多态位点, 本研究旨在探讨IL-6-634位点和IL-1β-31位点单核苷酸多态性与非吸烟女性肺癌易感性的关系, 并探讨环境危险因素与基因多态性交互作用对肺癌发病风险的影响。

**方法:**

采用病例-对照研究方法, 纳入非吸烟女性肺癌患者363例和对照370例。采用χ^2^检验比较各基因型、相关危险因素暴露在病例与对照之间分布的差异, 以比值比(odds ratio, OR)及其95%可信区间(confidence interval, CI)表示相对危险度。采用非条件*Logistic*回归分析多态基因型与肺癌间相关性及其与危险因素的交互作用。

**结果:**

IL-6-634位点携带CG基因型的个体发生肺癌危险升高(OR=1.61, 95%CI:1.19-2.19), CG或GG型携带者患肺癌的风险明显高于CC型的携带者(OR=1.48, 95%CI:1.10-1.98);IL-1β-31位点携带CT或TT型的个体肺癌的发病风险要低于CC携带者, 但差异无统计学意义。与携带IL-6-634 CC基因型且无油烟暴露者相比, 既有油烟暴露同时携带CG或GG基因型的个体发生肺癌的危险升高(OR=2.45, 95%CI:1.54-3.90), 而与携带CC型且无结核病史者相比, 既有结核病史并且携带CG或GG基因型的个体发生肺癌的危险升高(OR=2.44, 95%CI:1.05-5.66)。

**结论:**

*IL-6-634*基因多态性与非吸烟女性肺癌易感性相关且与油烟暴露和结核病史同时存在能增加患癌风险。

## Abstract (en)

**Background and objective:**

Interleukin-6 (IL-6) and interleukin-1β (IL-1β), known as multifunctional cytokines with high biological activity, play an important role in physiological and pathological responses such as inflammation, immune response and even tumors.There have been multiple polymorphism loci found in *IL-6* gene and *IL-1β* gene.The aim of this study is to investigate the relationship between IL-6-643 (C/G) and IL-1β-31 (C/T) polymorphisms and lung cancer risk among female non-smokers and explore the interaction effects on lung cancer risk between this two polymorphisms and potential risk factors such as cooking oil fumes exposure and history of tuberculosis.

**Methods:**

We performed a case-control study using 363 female lung cancer patients as cases and 370 healthy volunteers as controls.Genomic DNA was extracted from peripheral blood samples using classical phenol chloroform method.The genotyping of *IL-6*-634 or *IL-1β*-31 polymorphisms was performed using Taqman real time PCR technique by ABI7500.Two sided χ^2^ test was used to compare the distribution of the genotypes and risk factors between cases and controls.Unconditional *Logistic* regression analysis was performed to calculate the odds ratios (ORs) with 95% confidence intervals (CIs) for estimating the association between certain genotypes and lung cancer and exploring the interaction of risk factors and genetic polymorphisms.

**Results:**

The risk of lung cancer was significantly higher in those with IL-6-634 CG genotype than those with CC genotype (OR=1.61, 95%CI:1.19-2.19, *P*=0.002).The CG or GG genotype carriers had an elevated risk of lung cancer than CC genotype carriers (OR=1.48, 95%CI:1.10-1.98, *P*=0.01).No significant association was observed between *IL-1β-31* gene polymorphism and lung cancer risk.Compared with IL-6-634 CC genotype carriers with no cooking oil fumes exposure, a significant higher risk was found in individuals who were CG or GG genotype carriers with exposure to cooking oil fumes (OR=2.45, 95%CI:1.54-3.90).Compared with IL-6-634 CC genotype carriers with no history of tuberculosis, a significant elevated risk was found in individuals who were CG or GG genotype carriers with history of tuberculosis (OR=2.44, 95%CI:1.05-5.66).

**Conclusion:**

Our results indicated that IL-6-634 polymorphism was associated with the risk of lung cancer risk in female non-smokers.Individuals with both IL-6-634 CG or GG genotype and exposure to cooking oil fumes had a higher risk of lung cancer.Also individuals with both IL-6-634 CG or GG genotype and history of tuberculosis had an elevated risk of lung cancer.

肺癌是严重危害人类健康和生命的恶性肿瘤。尽管大规模流行病学调查已证实吸烟是确认的导致肺癌发生的最主要危险因素^[1, 2]^, 但是非吸烟肺癌患者的易感特征尚不明确, 尤其是女性肺癌多以非吸烟者居多。因此以非吸烟女性肺癌患者作为研究对象, 排除吸烟暴露因素的干扰, 探索肺癌的其他危险因素具有重要的意义。白介素-6(interleukin-6, IL-6)是白介素家族中具有多功能生物活性的细胞因子, 多种疾病可能都与*IL-6*基因调节异常有关, 包括炎症、自身免疫疾病和恶性肿瘤。研究报道^[3, 4]^IL-6-174、-572及-634位点存在单核苷酸多态性(single nucleotide polymorphism, SNP), 且与多种疾病发生有关。IL-6-634位点是一个C/G多态位点, 研究^[5, 6]^表明当该位点呈现C等位基因时, 外周血中IL-6水平明显升高, 该位点多态可能通过对mRNA转录及蛋白表达产生影响, 进而可能对肿瘤易感性产生影响。IL-1是一种前炎性细胞因子, 主要由单核巨噬细胞产生, 包括三个家族成员:IL-1α、IL-1β、IL-1βRa, 其中IL-1α、IL-1β为激动剂, 与广泛存在的靶细胞表面IL-1β受体结合, 产生生物学效应。IL-1β蛋白在炎症、肿瘤、排斥反应以及免疫性疾病等病理过程中常有异常表达, 并直接影响疾病的发生、发展和预后。研究^[7-9]^表明该基因上存在多个多态位点, 而其中位于启动子区的IL-1β-31、-511位点一直是国内外研究关注的热点。IL-1β-31位点位于*IL-1β*基因的启动子区, 研究^[10, 11]^报道该位点处于TATA盒序列内, C/T多态可能影响转录因子与基因的结合, 进而对正常的转录过程造成影响, 导致对肿瘤易感性的改变。

本研究分析沈阳地区非吸烟女性人群IL-6-634和IL-1β-31两个位点SNP与肺癌风险的关系, 并探索油烟暴露及结核病史与基因多态性的交互作用对肺癌发病风险的影响。

## 1 资料与方法

### 1.1 研究对象

本研究采用病例对照研究方法, 共纳入肺癌病例363例和健康对照370例。病例为2004年1月-2009年11月在辽宁省肿瘤医院进行治疗的非吸烟女性肺癌患者, 所有患者均经组织病理学确诊。对照选自于同期医院体检中心进行体检的健康女性, 按年龄(±5岁)与病例频数匹配。在收集血液标本的同时, 通过调查问卷的形式获取病例组与对照组的一般情况(姓名、年龄、民族、婚姻状况和受教育程度), 油烟暴露史、结核病史等危险因素暴露资料。非吸烟者指终生吸烟总数少于100支的个体; 烹饪油烟暴露史定义为从事烹饪15年以上, 每周煎或炸2次以上, 且烹调过程中经常出现眼部及咽喉部烟雾刺激感。所有观察对象均签署知情同意书并征得伦理委员会同意。

### 1.2 基因分型

采集外周静脉血后应用经典酚-氯仿法提取血液中的DNA, 应用Taqman荧光定量PCR基因分型技术进行IL-6-634和IL-1β-31位点基因多态性的检测, PCR使用的Taqman探针及引物由美国应用生物系统公司(ABI)设计合成, Taqman Master Mix购自美国应用生物系统公司。以SDS软件Allelic Discrimination程序进行基因型的分析, 通过检测同一基因不同等位基因所标记的FAM和VIC荧光强度, 判断待测样本的基因型。

### 1.3 统计学分析

以χ^2^检验比较各基因型、相关危险因素暴露在病例与对照之间分布的差异, 以比值比(odds ratio, OR)及其95%可信区间(confidence interval, CI)表示相对危险度。采用非条件*Logistic*回归分析多态基因型与肺癌间相关性及其与危险因素的交互作用。所有统计检验都为双侧检验, 检验水准α取0.05, 统计学分析均由SPSS 16.0软件完成。

## 2 结果

### 2.1 研究对象的基本情况

本研究共选取非吸烟女性肺癌病例363例, 对照370例, 所有观察对象的基本情况见表 1。病例组与对照组间在年龄、受教育程度方面比较差异无统计学意义(*P* > 0.05);危险因素暴露的比较中发现, 病例组中有油烟暴露的个体和有结核病史的个体所占比例均高于对照组, 差异有统计学意义, 计算比值比后表明有油烟暴露的个体患癌风险是无油烟暴露个体的1.81倍(95%CI:1.32-2.49, *P* < 0.001), 有结核病史的个体患癌风险是无结核病史个体的1.84倍(95%CI:1.09-3.11, *P*=0.022), 提示油烟暴露和结核病史可能是非吸烟女性肺癌的危险因素。

**1:** 病例组与对照组的基本情况 Demographics of lung cancer patients and healthy controls

	Cases (%)	Controls (%)	*P*
Female	363	370	
Age (yr)	55.79±11.79	56.46±12.06	0.448
Education			0.799
Never	34 (9.4)	32 (8.6)	
Elementary school	171 (47.1)	188 (50.8)	
Junior school	108 (29.8)	102 (27.6)	
Senior school and upwards	50 (13.8)	48 (13.0)	
Histology			
Adenocarcinoma	248 (68.3)	-	-
Squamous cell carcinoma	58 (16.0)	-	-
Other types	57 (15.7)	-	-
Exposure to cooking oil fumes			< 0.001^*^
Yes	136 (37.5)	92 (24.9)	
No	227 (62.5)	278 (75.1)	
History of tuberculosis			0.022^*^
Yes	41 (11.3)	24 (6.5)	
No	322 (88.7)	346 (93.5)	
^*^Statistically significant.			

### 2.2 IL-6-634和IL-1β-31位点多态性与非吸烟女性肺癌危险性的关系

IL-6-634位点(rs1800796)为C→G多态, χ^2^拟合优度检验表明病例组与对照组基因型分布均符合*Hardy-Weinberg*平衡定律(χ^2^=2.608, *P*=0.271;χ^2^=2.696, *P*=0.260), 病例组及对照组基因型频率见表 2。*Logistic*回归分析结果表明, 携带CG基因型的个体发生肺癌的危险性是携带CC基因型个体的1.61倍(95%CI:1.19-2.19, *P*=0.002);至少携带一个突变等位基因G的个体(CG/GG)患肺癌的风险同样高于CC基因型的个体(OR=1.48, 95%CI:1.10-1.98, *P*=0.010)。IL-1β-31位点(rs1143627)为C→T多态, χ^2^拟合优度检验表明病例组与对照组基因型分布均符合*Hardy-Weinberg*平衡定律(χ^2^=0.297, *P*=0.862;χ^2^=0.294, *P*=0.863)。*Logistic*回归分析结果并未发现不同变异基因型与非吸烟女性肺癌易感性存在有统计学意义的关联。

**2:** *IL-6*-634和*IL-1β*-31位点多态性与非吸烟女性肺癌危险性的关系 Association between *IL-6*-634 and *IL-1β*-31 polymorphisms and the risk of lung cancer risk in female non-smokers

Genotypes	Cases (%)	Controls (%)	OR (95%CI)^a^	*P* value
*IL-6-634*				
CC	193 (53.2)	233 (63.0)	1.00 (ref)	
CG	156 (43.0)	117 (31.6)	1.61 (1.19-2.19)	0.002^*^
GG	14 (3.9)	20 (5.4)	0.85 (0.42-1.72)	0.642
CG/GG	170 (46.8)	137 (37.0)	1.48 (1.10-1.98)	0.010^*^
*IL-1β-31*				
CC	89 (24.5)	80 (21.6)	1.00 (ref)	
CT	194 (53.4)	199 (53.8)	0.88 (0.61-1.26)	0.473
TT	80 (22.0)	91 (24.6)	0.79 (0.52-1.21)	0.279
CT/TT	274 (75.5)	290 (78.4)	0.85(0.60-1.20)	0.352
a:OR; ^*^:Statistically significant; OR:odds ratio; CI:confidence interval.				

### 2.3 IL-6-634位点多态性与暴露危险因素交互作用

本研究进一步分析了IL-6-634位点多态性与油烟暴露或结核病史联合作用对肺癌发病风险的影响(表 3)。叉生分析结果显示以携带CC基因型且无油烟暴露者为参照, 单纯携带CG或GG基因型者发生肺癌的风险升高(OR=1.64, 95%CI:1.14-2.35), 单纯有油烟暴露者发生肺癌的风险同样增高(OR=1.97, 95%CI:1.29-3.01), 既有油烟暴露同时携带CG或GG基因型发生肺癌的危险性明显升高(OR=2.45, 95%CI:1.54-3.90), 均提示IL-6-634突变基因型CG或GG与油烟暴露联合作用可能增加非吸烟女性肺癌发生的风险, *Logistic*回归结果中油烟暴露与基因型交互项显示这种联合作用不存在统计学意义(*P*=0.329), 说明油烟暴露与突变基因型之间可能不存在相乘交互作用; 接下来我们以相加交互作用模型计算了交互作用指数并进行了参数估计, 结果显示油烟暴露与突变基因型之间可能不存在相加交互作用(SI=0.85, 95%CI:0.36-2.00);与携带CC基因型且无结核病史者相比, 携带CG或GG基因型者发生肺癌危险升高(OR=1.52, 95%CI:1.11-2.08), 既有结核病史并且携带CG或GG基因型者患癌危险明显升高(OR=2.44, 95%CI:1.05-5.66), 结果提示IL-6-634突变基因型CG或GG联合结核病史可能增加肺癌发生的危险性, *Logistic*回归结果中结核病史与基因型交互项显示这种联合作用不存在统计学意义(*P*=0.628), 说明结核病史与突变基因型之间可能不存在相乘交互作用; 以相加交互作用模型计算交互作用指数并进行参数估计结果显示, 结核病史与突变基因型之间可能不存在相加交互作用(SI=0.90, 95%CI:0.18-4.53)。

**3:** IL-6-634基因多态与油烟暴露及结核病史的交互作用对非吸烟女性肺癌发病风险的影响 Interaction of IL-6-634 polymorphism and cooking oil fumes exposure and history of tuberculosis on risk of lung cancer in female non-smokers

Variables	Genotypes	Cases	Controls	OR (95%CI)	*P*
Exposure to cooking oil fumes					
No	CC	119	179	1.00 (ref)	
No	CG+GG	108	99	1.64 (1.14-2.35)^a^	0.007^*^
Yes	CC	74	54	1.97 (1.29-3.01)^a^	0.002^*^
Yes	CG+GG	62	38	2.45 (1.54-3.90)^a^	< 0.001^*^
History of tuberculosis					
No	CC	169	218	1.00 (ref)	
No	CG+GG	153	128	1.52 (1.11-2.08)^b^	0.009^*^
Yes	CC	24	15	1.83 (0.92-3.64)^b^	0.073
Yes	CG+GG	17	9	2.44 (1.05-5.66)^b^	0.037^*^
^a^:OR was adjusted for age and history of tuberculosis; ^b^:OR was adjusted for age and cooking oil fumes exposure; ^*^:Statistically significant.					

## 3 讨论

肺癌是世界范围内常见的恶性肿瘤之一, 研究认为肺癌的发生是一个多基因参与、多因素共同作用的多阶段过程, 除与吸烟、环境污染、职业等暴露因素存在密切联系外, 还与个体对肺癌的遗传易感性有关。已知SNP是引起个体对肺癌遗传易感性的主要原因^[12]^, 能够影响烟草及其他环境致癌物的代谢和解毒、DNA损伤修复、细胞周期的调控以及其他细胞应答反应。

*IL-6*基因编码的蛋白在肿瘤的发生发展过程中有重要的作用, 能作为自分泌和旁分泌的生长因子刺激或抑制肿瘤细胞生长。不少类型的肿瘤细胞都能产生IL-6, Yanagawa等^[13]^报道在肺癌患者的胸腔积液中检测到了IL-6, 有些研究也表明某些肺癌细胞株可产生IL-6;Arias等^[14]^检测了22例肺癌患者和8例正常人支气管肺泡灌洗液中IL-6水平, 结果发现肺癌患者的IL-6水平明显高于对照组。*IL-6*基因位于染色体7p21区域, 有报道^[15, 16]^称其SNP与前列腺癌、乳腺癌发生相关联。李岩等^[17]^对中国北方地区人群非小细胞肺癌易感性研究发现, IL-6启动子区-572位点SNP与肺癌易感性相关, 风险降低主要发生在肺鳞癌, 而与肺腺癌无关。本研究中IL-6-634位点的研究结果表明, 对照人群突变杂合型CG和突变纯合型GG的基因型频率分别为31.6%、5.4%, 突变等位基因G的频率为21.2%, 与Seow等^[18]^(34.0%, 6.2%)和Lim等^[19]^(32.2%, 5.3%)结果基本一致; 而病例组基因型频率分布与对照组相比有明显差异, 且携带突变型CG或GG者患肺癌的风险要明显高于携带野生型CC者, 提示IL-6-634位点SNP可能是非吸烟女性肺癌发生的独立危险因素, OR值为1.61, 但与Seow等的研究结果不一致(OR=1.1, 95%CI:0.7-1.8), 虽然都是选择非吸烟人群, 但是Seow等选择的是定居在新加坡的中国女性, 可能在环境暴露因素方面存在差异。

*IL-1β*基因位于人染色体2q13区域, 其调控序列存在与蛋白浓度相关的SNPs位点, 直接决定了IL-1β蛋白的表达水平, 进而影响疾病的发生、发展和预后。有研究发现其中位于启动子区的IL-1β-31、-511位点SNP与肿瘤发生相关, Zienolddiny等^[20]^分析IL-1β-31、-511位点与非小细胞肺癌危险的联系, 研究指出携带-31T等位基因和-511C等位基因可明显增加患肺癌的危险性, 携带-31TT和-511CC基因型患肺癌的风险分别是携带野生型的2.39和2.51倍。本研究选择-31位点SNP探讨其与非吸烟女性肺癌的关联, 结果提示携带CT或TT突变型者肺癌的发病风险要低于CC携带者, 但差异无统计学意义, 这与Zienolddiny等的研究结果相矛盾, 可能是由于本研究选取的是非吸烟女性, 排除了吸烟因素的混杂; 另外, 本次对照人群的CC、CT、TT基因型频率分别为21.6%、53.8%、24.6%, 与Zienolddiny等^[20]^研究的对照人群频率存在一定差异, 也可能与该位点基因多态在不同种族之间分布存在差异有关。

近年来女性肺癌的发病率急速上升, 尤其在我国, 女性肺癌发病率是日本、高加索人群的两倍甚至更高, 但我国女性的主动吸烟率远低于欧美, 绝大多数肺癌的发生不能归因于吸烟, 可能是由环境致癌物的暴露, 慢性呼吸系统疾病引起^[21]^。目前女性肺癌和油烟暴露的关系受到广泛的关注, 已有报道^[22]^称烹调时高温加热食用油产生的油烟中发现了多环芳烃、芳香胺等致癌物。并且有研究^[23]^发现结核感染与肺癌发生可能相关, 在结核分枝杆菌感染的肺组织中发现巨噬细胞可以产生一种与表皮生长因子功能相似的上皮调节蛋白, 可能造成鳞状上皮化生并导致肺癌的发生。本研究结果发现有油烟暴露的女性患肺癌的风险是无油烟暴露者的1.81倍(95%CI:1.32-2.49, *P* < 0.001);此外还发现有结核病史者发生肺癌的风险是无结核病史者的1.84倍(95%CI:1.09-3.11, *P*=0.022)。以上结果均提示油烟暴露和结核病史可能是非吸烟女性肺癌发生的危险因素。携带IL-6-634杂合CG基因型同时伴有油烟暴露时, 比单独油烟暴露或单独携带IL-6-634杂合CG基因型时发生肺癌的风险要明显增加, 提示我们*IL-6*-634基因多态性与油烟暴露可能存在交互作用, 既有结核病史并且携带CG或GG基因型者患肺癌的风险也高于单纯携带CG或GG型者或单纯有结核病史者, 提示结核病史与IL-6-634杂合基因型也存在交互作用。但本研究交互作用并未得到任何有统计学意义的结果, 因为本研究的样本量不大, 可能会影响交互作用统计学检验的效能; 另外由于未考虑其它未知的可能的混杂因素的作用, 我们得到的交互作用指数SI都是小于1的, 所以也不能简单认为油烟暴露或结核病史与突变基因型之间相互独立, 仍需要后续研究进一步进行验证。

本研究也存在一定的局限性, 选取同期医院体检中心进行体检的健康女性, 可能从一定程度上造成偏倚, 这些健康女性来自某个有条件参加体检的阶层, 并且可能与病例在居住地分布上都会有差别, 因此在评价环境暴露与患癌风险关联时得出的关联强度可能会被夸大; 另外本研究样本量不大, 可能在评估环境-基因型的交互作用时降低检验效能, 对结果产生影响。

综上所述, 本研究相比较以往研究扩大了样本量并对非吸烟女性肺癌进行了针对性的研究, 发现油烟暴露、结核病史和*IL-6*-634基因多态均是非吸烟女性肺癌发生的危险因素, 油烟暴露与IL-6-634突变基因型同时存在可能增加非吸烟女性患肺癌的风险, IL-6-634突变基因型联合结核病史同样能够增加女性肺癌发生的危险性。*IL-1β*-31基因多态性可能与非吸烟女性肺癌发生无关。以上结果有助于后续研究者了解沈阳市非吸烟女性肺癌发生的危险因素, 能够更好地对肺癌高危人群进行筛查、早诊和治疗。在今后的研究中我们将继续收集标本, 在肺癌不同组织学类型病例中继续对结果进行验证, 另外*IL-6*和*IL-1β*基因多态性与其他炎性细胞因子多态性的交互作用也值得进一步研究。
